# 1-(2,4-Difluoro­phen­yl)-2-(1*H*-1,2,4-triazol-1-yl)ethanol

**DOI:** 10.1107/S1600536812001110

**Published:** 2012-01-18

**Authors:** Dong-liang Liu, Chen Li, Xin Tian, Song Li, Tao Xiao

**Affiliations:** a Department of Applied Chemistry, College of Science, Nanjing University of Technology, Nanjing 210009, People’s Republic of China

## Abstract

In the title compound, C_10_H_9_F_2_N_3_O, the dihedral angle between the rings is 22.90 (4)°. In the crystal, C—H⋯F and O—H⋯N hydrogen bonds link the mol­ecules into chains along [010].

## Related literature

For related compounds containing a 2-(1*H*-1,2,4-triazol-1-yl)-1-phenyl­ethanol fragment, see: Bu *et al.* (2000 ▶). For related structures, see: Tao *et al.* (2007 ▶); Liu *et al.* (2011 ▶); Yu *et al.* (2011 ▶). For standard bond lengths, see: Allen *et al.* (1987 ▶).
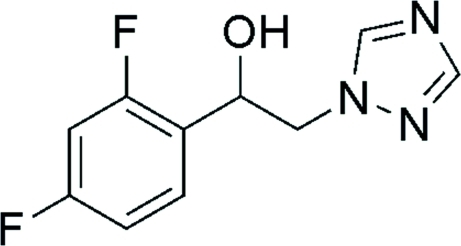


## Experimental

### 

#### Crystal data

C_10_H_9_F_2_N_3_O
*M* *_r_* = 225.20
Monoclinic, 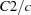

*a* = 14.261 (3) Å
*b* = 5.6150 (11) Å
*c* = 25.823 (5) Å
β = 94.84 (3)°
*V* = 2060.4 (7) Å^3^
*Z* = 8
Mo *K*α radiation
μ = 0.12 mm^−1^
*T* = 293 K
0.30 × 0.10 × 0.10 mm

#### Data collection

Enraf–Nonius CAD-4 diffractometer
Absorption correction: ψ scan (North *et al.*, 1968 ▶) *T*_min_ = 0.964, *T*_max_ = 0.988
1969 measured reflections
1886 independent reflections
1059 reflections with *I* > 2σ(*I*)
*R*_int_ = 0.035
3 standard reflections every 200 reflections intensity decay: 1%

#### Refinement

*R*[*F* ^2^ > 2σ(*F* ^2^)] = 0.051
*wR*(*F* ^2^) = 0.143
*S* = 1.01
1886 reflections
148 parameters
3 restraints
H atoms treated by a mixture of independent and constrained refinement
Δρ_max_ = 0.17 e Å^−3^
Δρ_min_ = −0.15 e Å^−3^

### 

Data collection: *CAD-4 EXPRESS* (Enraf–Nonius, 1994 ▶); cell refinement: *CAD-4 EXPRESS*; data reduction: *XCAD4* (Harms & Wocadlo, 1995 ▶); program(s) used to solve structure: *SHELXS97* (Sheldrick, 2008 ▶); program(s) used to refine structure: *SHELXL97* (Sheldrick, 2008 ▶); molecular graphics: *PLATON* (Spek, 2009 ▶); software used to prepare material for publication: *SHELXTL* (Sheldrick, 2008 ▶).

## Supplementary Material

Crystal structure: contains datablock(s) I, global. DOI: 10.1107/S1600536812001110/zq2149sup1.cif

Structure factors: contains datablock(s) I. DOI: 10.1107/S1600536812001110/zq2149Isup2.hkl

Supplementary material file. DOI: 10.1107/S1600536812001110/zq2149Isup3.cml

Additional supplementary materials:  crystallographic information; 3D view; checkCIF report

## Figures and Tables

**Table 1:** Hydrogen-bond geometry (Å, °)

*D*—H⋯*A*	*D*—H	H⋯*A*	*D*⋯*A*	*D*—H⋯*A*
O—H0*A*⋯N3^i^	0.83 (3)	1.98 (3)	2.794 (3)	169 (3)
C8—H8*B*⋯F2^ii^	0.97	2.46	3.388 (4)	159

Symmetry codes: (i) 
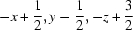
; (ii) 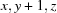.

**Fig. 1.**
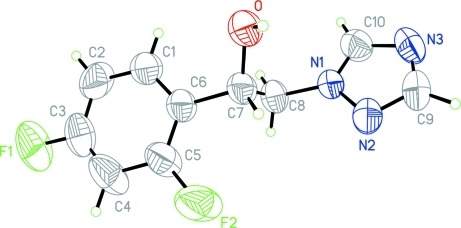
The molecular structure of the title compound. Displacement ellipsoids are drawn at the 50% probability level.

**Fig. 2.**
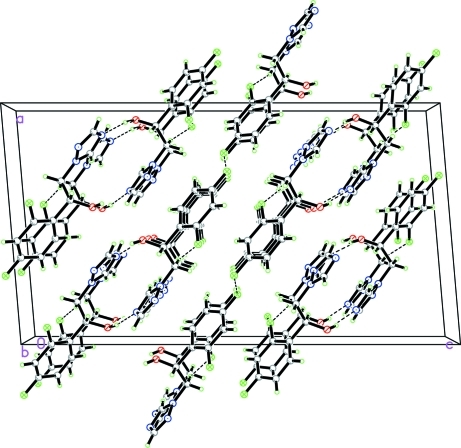
A packing diagram of the title compound. Hydrogen bonds are shown as dashed lines.

## supplementary crystallographic information

### Comment

The title compound, C_10_H_9_O_1_N_3_F_2_, is the key intermediate in the synthesis of a new kind of antifungal drug (Bu *et al.*, 2000). We previously reported the crystal structures of similar compounds (Tao *et al.*, 2007; Liu *et al.*, 2011; Yu *et al.*, 2011). The X-ray diffraction study has been carried out in order to elucidate the molecular conformation.We report here its crystal structure (Fig. 1). The bond lengths and angles are within normal ranges (Allen *et al.*, 1987). The dihedral angle between the ring A (N1/N2/C9—C11) and B (C1—C6) is 22.90 (4)°. In the crystal, intermolecular C—H···F and O—H···N hydrogen bonds link the molecules into one-dimensional [010] chains (Fig. 2).

### Experimental

A mixture of 1-(2,4-difluorophenyl)-2-(1*H*-1,2,4-triazol-1-yl)ethanone (2.25 g, 10 mol), sodium borohydride (0.756 g, 20 mmol) and 30 ml dry ethanol was refluxed for 3 h. After solvent evaporation, the mixture was neutralized with dilute hydrochloric acid and then refluxed for 30 min. After the mixture was cooled, the solution was alkalinized with sodium hydroxide, the precipitate collected and recrystallized with ethanol, and a yellow deposit was obtain (m.p. 395–396 K). Crystals suitable for X-ray analysis were obtained by dissolving the crude product (1.0 g) in ethanol (30 ml) and then allowing the solution to evaporate slowly at room temperature for about 7 d.

### Refinement

The H atom of the hydroxy group was located in a Fourier difference map and freely refined with *U*_iso_(H) = 1.5*U*_eq_(O). The other H atoms were positioned geometrically with C—H = 0.93 Å (aromatic) and 0.97 Å (methylene) and constrained to ride on their parent atoms with *U*_iso_(H) = 1.2*U*_eq_(C).

### Crystal data

**Table:**

C_10_H_9_F_2_N_3_O	*F*(000) = 928
*M**_r_* = 225.20	*D*_x_ = 1.452 Mg m^−^^3^
Monoclinic, *C*2/*c*	Melting point: 395 K
Hall symbol: -C 2yc	Mo *K*α radiation, λ = 0.71073 Å
*a* = 14.261 (3) Å	Cell parameters from 25 reflections
*b* = 5.6150 (11) Å	θ = 9–13°
*c* = 25.823 (5) Å	µ = 0.12 mm^−^^1^
β = 94.84 (3)°	*T* = 293 K
*V* = 2060.4 (7) Å^3^	Prism, colourless
*Z* = 8	0.30 × 0.10 × 0.10 mm

### Data collection

**Table:**

Enraf–Nonius CAD-4 diffractometer	1059 reflections with *I* > 2σ(*I*)
Radiation source: fine-focus sealed tube	*R*_int_ = 0.035
graphite	θ_max_ = 25.4°, θ_min_ = 1.6°
ω/2θ scans	*h* = 0→17
Absorption correction: ψ scan (North *et al.*, 1968)	*k* = 0→6
*T*_min_ = 0.964, *T*_max_ = 0.988	*l* = −31→30
1969 measured reflections	3 standard reflections every 200 reflections
1886 independent reflections	intensity decay: 1%

### Refinement

**Table:**

Refinement on *F*^2^	Primary atom site location: structure-invariant direct methods
Least-squares matrix: full	Secondary atom site location: difference Fourier map
*R*[*F*^2^ > 2σ(*F*^2^)] = 0.051	Hydrogen site location: inferred from neighbouring sites
*wR*(*F*^2^) = 0.143	H atoms treated by a mixture of independent and constrained refinement
*S* = 1.00	*w* = 1/[σ^2^(*F*_o_^2^) + (0.070*P*)^2^] where *P* = (*F*_o_^2^ + 2*F*_c_^2^)/3
1886 reflections	(Δ/σ)_max_ < 0.001
148 parameters	Δρ_max_ = 0.17 e Å^−^^3^
3 restraints	Δρ_min_ = −0.14 e Å^−^^3^

### Special details

**Table:**

Geometry. All e.s.d.'s (except the e.s.d. in the dihedral angle between two l.s. planes) are estimated using the full covariance matrix. The cell e.s.d.'s are taken into account individually in the estimation of e.s.d.'s in distances, angles and torsion angles; correlations between e.s.d.'s in cell parameters are only used when they are defined by crystal symmetry. An approximate (isotropic) treatment of cell e.s.d.'s is used for estimating e.s.d.'s involving l.s. planes.
Refinement. Refinement of *F*^2^ against ALL reflections. The weighted *R*-factor *wR* and goodness of fit *S* are based on *F*^2^, conventional *R*-factors *R* are based on *F*, with *F* set to zero for negative *F*^2^. The threshold expression of *F*^2^ > σ(*F*^2^) is used only for calculating *R*-factors(gt) *etc*. and is not relevant to the choice of reflections for refinement. *R*-factors based on *F*^2^ are statistically about twice as large as those based on *F*, and *R*- factors based on ALL data will be even larger.

### Fractional atomic coordinates and isotropic or equivalent isotropic displacement parameters (Å^2^)

**Table:**

	*x*	*y*	*z*	*U*_iso_*/*U*_eq_	
O	0.06636 (12)	0.1378 (4)	0.69742 (7)	0.0730 (6)	
H0A	0.080 (2)	0.037 (5)	0.7204 (11)	0.088*	
N1	0.25384 (13)	0.1969 (4)	0.66832 (7)	0.0604 (6)	
F1	−0.20749 (13)	−0.0256 (4)	0.49804 (8)	0.1263 (8)	
C1	−0.05951 (18)	0.1696 (6)	0.60844 (11)	0.0895 (10)	
H1A	−0.0572	0.2907	0.6331	0.107*	
F2	0.07930 (15)	−0.3219 (4)	0.57292 (8)	0.1337 (9)	
N2	0.30591 (16)	0.0018 (5)	0.68043 (10)	0.0848 (8)	
C2	−0.13473 (19)	0.1639 (7)	0.57217 (12)	0.0971 (11)	
H2B	−0.1831	0.2752	0.5718	0.117*	
N3	0.36133 (15)	0.3146 (5)	0.72719 (8)	0.0787 (7)	
C3	−0.1347 (2)	−0.0224 (7)	0.53496 (12)	0.0873 (10)	
C4	−0.0688 (2)	−0.1827 (6)	0.53470 (12)	0.1006 (11)	
H4A	−0.0714	−0.3041	0.5101	0.121*	
C5	0.0087 (2)	−0.1631 (5)	0.57445 (11)	0.0821 (9)	
C6	0.01250 (17)	0.0092 (5)	0.61091 (9)	0.0617 (7)	
C7	0.09468 (17)	0.0301 (5)	0.65216 (9)	0.0604 (7)	
H7A	0.1195	−0.1292	0.6607	0.072*	
C8	0.17176 (16)	0.1809 (5)	0.63143 (9)	0.0686 (7)	
H8A	0.1898	0.1118	0.5993	0.082*	
H8B	0.1477	0.3397	0.6238	0.082*	
C9	0.36890 (19)	0.0821 (6)	0.71531 (12)	0.0851 (9)	
H9A	0.4161	−0.0143	0.7311	0.102*	
C10	0.2871 (2)	0.3760 (5)	0.69662 (11)	0.0760 (8)	
H10A	0.2613	0.5282	0.6952	0.091*	

### Atomic displacement parameters (Å^2^)

**Table:**

	*U*^11^	*U*^22^	*U*^33^	*U*^12^	*U*^13^	*U*^23^
O	0.0753 (11)	0.0893 (15)	0.0522 (10)	0.0145 (11)	−0.0081 (8)	0.0017 (10)
N1	0.0569 (11)	0.0675 (13)	0.0548 (12)	0.0103 (11)	−0.0083 (9)	−0.0079 (11)
F1	0.1093 (14)	0.1456 (18)	0.1112 (15)	−0.0102 (13)	−0.0652 (12)	0.0063 (13)
C1	0.0722 (17)	0.121 (3)	0.0707 (18)	0.0322 (19)	−0.0212 (14)	−0.0251 (19)
F2	0.1684 (18)	0.0919 (14)	0.1278 (16)	0.0621 (14)	−0.0645 (14)	−0.0378 (12)
N2	0.0710 (14)	0.0808 (16)	0.0969 (18)	0.0165 (13)	−0.0257 (13)	−0.0199 (14)
C2	0.0697 (17)	0.141 (3)	0.0767 (19)	0.039 (2)	−0.0157 (15)	−0.005 (2)
N3	0.0761 (15)	0.095 (2)	0.0616 (13)	−0.0125 (14)	−0.0126 (11)	−0.0080 (13)
C3	0.0747 (19)	0.104 (2)	0.078 (2)	−0.0099 (19)	−0.0289 (16)	0.0163 (19)
C4	0.127 (3)	0.073 (2)	0.091 (2)	−0.002 (2)	−0.051 (2)	−0.0071 (18)
C5	0.101 (2)	0.0584 (17)	0.0804 (19)	0.0165 (17)	−0.0334 (16)	−0.0083 (15)
C6	0.0612 (14)	0.0680 (16)	0.0533 (14)	0.0083 (14)	−0.0098 (11)	0.0007 (13)
C7	0.0643 (14)	0.0636 (16)	0.0506 (13)	0.0115 (12)	−0.0114 (11)	−0.0024 (12)
C8	0.0683 (15)	0.0824 (18)	0.0519 (13)	0.0105 (14)	−0.0147 (11)	0.0038 (14)
C9	0.0644 (17)	0.102 (3)	0.084 (2)	0.0084 (17)	−0.0214 (15)	−0.0041 (19)
C10	0.0809 (18)	0.0693 (18)	0.0763 (19)	−0.0009 (15)	−0.0025 (15)	−0.0094 (16)

### Geometric parameters (Å, °)

**Table:**

O—C7	1.405 (3)	N3—C10	1.312 (3)
O—H0A	0.83 (3)	N3—C9	1.347 (4)
N1—C10	1.308 (3)	C3—C4	1.302 (4)
N1—N2	1.345 (3)	C4—C5	1.448 (3)
N1—C8	1.448 (3)	C4—H4A	0.9300
F1—C3	1.349 (3)	C5—C6	1.348 (3)
C1—C6	1.363 (3)	C6—C7	1.520 (3)
C1—C2	1.364 (3)	C7—C8	1.520 (4)
C1—H1A	0.9300	C7—H7A	0.9800
F2—C5	1.348 (3)	C8—H8A	0.9700
N2—C9	1.298 (3)	C8—H8B	0.9700
C2—C3	1.420 (4)	C9—H9A	0.9300
C2—H2B	0.9300	C10—H10A	0.9300
			
C7—O—H0A	104 (2)	C5—C6—C1	117.1 (2)
C10—N1—N2	109.2 (2)	C5—C6—C7	121.9 (2)
C10—N1—C8	130.5 (2)	C1—C6—C7	121.0 (2)
N2—N1—C8	120.1 (2)	O—C7—C8	108.7 (2)
C6—C1—C2	124.2 (3)	O—C7—C6	110.93 (19)
C6—C1—H1A	117.9	C8—C7—C6	109.5 (2)
C2—C1—H1A	117.9	O—C7—H7A	109.2
C9—N2—N1	102.4 (2)	C8—C7—H7A	109.2
C1—C2—C3	115.9 (3)	C6—C7—H7A	109.2
C1—C2—H2B	122.0	N1—C8—C7	111.78 (19)
C3—C2—H2B	122.0	N1—C8—H8A	109.3
C10—N3—C9	101.2 (2)	C7—C8—H8A	109.3
C4—C3—F1	120.0 (3)	N1—C8—H8B	109.3
C4—C3—C2	123.6 (3)	C7—C8—H8B	109.3
F1—C3—C2	116.4 (3)	H8A—C8—H8B	107.9
C3—C4—C5	116.9 (3)	N2—C9—N3	115.5 (3)
C3—C4—H4A	121.6	N2—C9—H9A	122.2
C5—C4—H4A	121.6	N3—C9—H9A	122.2
F2—C5—C6	120.6 (2)	N1—C10—N3	111.7 (3)
F2—C5—C4	117.2 (2)	N1—C10—H10A	124.1
C6—C5—C4	122.2 (3)	N3—C10—H10A	124.1
			
C10—N1—N2—C9	−1.0 (3)	C2—C1—C6—C7	179.1 (3)
C8—N1—N2—C9	−177.4 (2)	C5—C6—C7—O	−152.5 (3)
C6—C1—C2—C3	−0.7 (5)	C1—C6—C7—O	29.4 (4)
C1—C2—C3—C4	0.9 (5)	C5—C6—C7—C8	87.5 (3)
C1—C2—C3—F1	−178.2 (3)	C1—C6—C7—C8	−90.6 (3)
F1—C3—C4—C5	177.9 (3)	C10—N1—C8—C7	−108.9 (3)
C2—C3—C4—C5	−1.1 (5)	N2—N1—C8—C7	66.5 (3)
C3—C4—C5—F2	−177.0 (3)	O—C7—C8—N1	61.5 (3)
C3—C4—C5—C6	1.3 (5)	C6—C7—C8—N1	−177.2 (2)
F2—C5—C6—C1	177.0 (3)	N1—N2—C9—N3	0.5 (4)
C4—C5—C6—C1	−1.2 (5)	C10—N3—C9—N2	0.2 (4)
F2—C5—C6—C7	−1.1 (4)	N2—N1—C10—N3	1.3 (3)
C4—C5—C6—C7	−179.3 (3)	C8—N1—C10—N3	177.1 (2)
C2—C1—C6—C5	0.9 (5)	C9—N3—C10—N1	−0.9 (3)

### Hydrogen-bond geometry (Å, °)

**Table:**

*D*—H···*A*	*D*—H	H···*A*	*D*···*A*	*D*—H···*A*
O—H0A···N3^i^	0.83 (3)	1.98 (3)	2.794 (3)	169 (3)
C8—H8B···F2^ii^	0.97	2.46	3.388 (4)	159

Symmetry codes: (i) −*x*+1/2, *y*−1/2, −*z*+3/2; (ii) *x*, *y*+1, *z*.
